# Bioactive Lipidic Extracts from Octopus (*Paraoctopus limaculatus*): Antimutagenicity and Antiproliferative Studies

**DOI:** 10.1155/2013/273582

**Published:** 2013-01-16

**Authors:** Carolina Moreno-Félix, Griselda Wilson-Sánchez, Susana-Gabriela Cruz-Ramírez, Carlos Velázquez-Contreras, Maribel Plascencia-Jatomea, Ana Acosta, Lorena Machi-Lara, María-Lourdes Aldana-Madrid, Josafat-Marina Ezquerra-Brauer, Fernando Rocha-Alonzo, Armando Burgos-Hernández

**Affiliations:** ^1^Departamento de Investigación y Posgrado en Alimentos, Universidad de Sonora, Apartado Postal 1658, 83000 Hermosillo, SON, Mexico; ^2^Departamento de Ciencias Químico-Biológicas, Universidad de Sonora, 83000 Hermosillo, SON, Mexico; ^3^Rubio Pharma y Asociados, S.A. de C.V., Blvd. García Morales No. 330, 83210 Hermosillo, SON, Mexico; ^4^Departamento de Investigación en Polímeros y Materiales, Universidad de Sonora, 83000 Hermosillo, SON, Mexico

## Abstract

Fractions from an organic extract from fresh octopus (*Paraoctopus limaculatus*) were studied for biological activities such as antimutagenic and antiproliferative properties using *Salmonella* tester strains TA98 and TA100 with metabolic activation (S9) and a cancer cell line (B-cell lymphoma), respectively. A chloroform extract obtained from octopus tentacles was sequentially fractionated using thin layer chromatography (TLC), and each fraction was tested for antimutagenic and antiproliferative activities. Organic extract reduced the number of revertants caused by aflatoxin B_1_ showing a dose-response type of relationship. Sequential TLC fractionation of the active extracts produced several antimutagenic and/or antiproliferative fractions. Based on the results obtained, the isolated fractions obtained from octopus contain compounds with chemoprotective properties that reduce the mutagenicity of AFB_1_ and proliferation of cancer cell lines.

## 1. Introduction

As food toxicology develops, more kinds of chemical mutagens and carcinogens are found to be present in foods. Many are food contaminants such as mycotoxins and pesticides, but others are present in food as a consequence of technological processes applied to them (e.g., pyrolysates, nitrosamines, polycyclic aromatic hydrocarbons, etc.) [1]. However, a great variety of compounds that are natural constituents of foods have been found to show properties that are beneficial for the consumer. Among the kinds of “functional” compounds found in food are anticholesterolemic compounds, antioxidants, antivirals, and so forth. The antimutagenic and antiproliferative agents can be considered in this group of bioactive compounds. These biologically active compounds include thiocyanates, indole-3-carbinol, allium compounds, linoleic acid, conjugated linoleic acid, polyunsaturated fatty acids, caffeic acid phenethyl ester (CAPE), and flavonoids such as pinocembrin, rutin, naringenin, and hesperetin, respectively [2].

Unsaturated fatty acids, especially the *ω*-3 and *ω*-6, are components of the lipid fraction of marine organisms that have been associated to the prevention of chronic degenerative diseases such as cardiovascular diseases and cancer [3–5]. Structurally, *ω*-3 and *ω*-6 fatty acids are similar to linoleic and linolenic acids, to which antimutagenicity and anticarcinogenicity have been reported [3, 6, 7]. Based on these facts, the present investigation focused on the detection of antimutagenic and antiproliferative compounds in octopus, an economically important and highly consumed seafood worldwide.

## 2. Materials and Methods

### 2.1. Testing Species

Octopus (*Paraoctopus limaculatus*) was obtained from Bahía de Kino, SON, México (approximately 2000 km northwest México City, México) and transported in ice to the University of Sonora Seafood Laboratory. Edible portions from octopus were separated, fresh packed, and stored at −25°C until further analysis.

*Octopus Extract*. A 100 g portion of octopus muscle and 5 parts of CHCl_3_ were homogenized in a blender at high speed for 1 min. Resulting mix was poured into a flask and agitated during 30 min with the aid of a Wrist Action Burrel Shaker (Burrel Corporation, Pittsburg, PA). The mix was filtered through Whatman no. 4 filter paper with vacuum, and the filtrate was evaporated to dryness under N_2_ stream.

### 2.2. Fractionation of Octopus Extract

**Figure 1:**
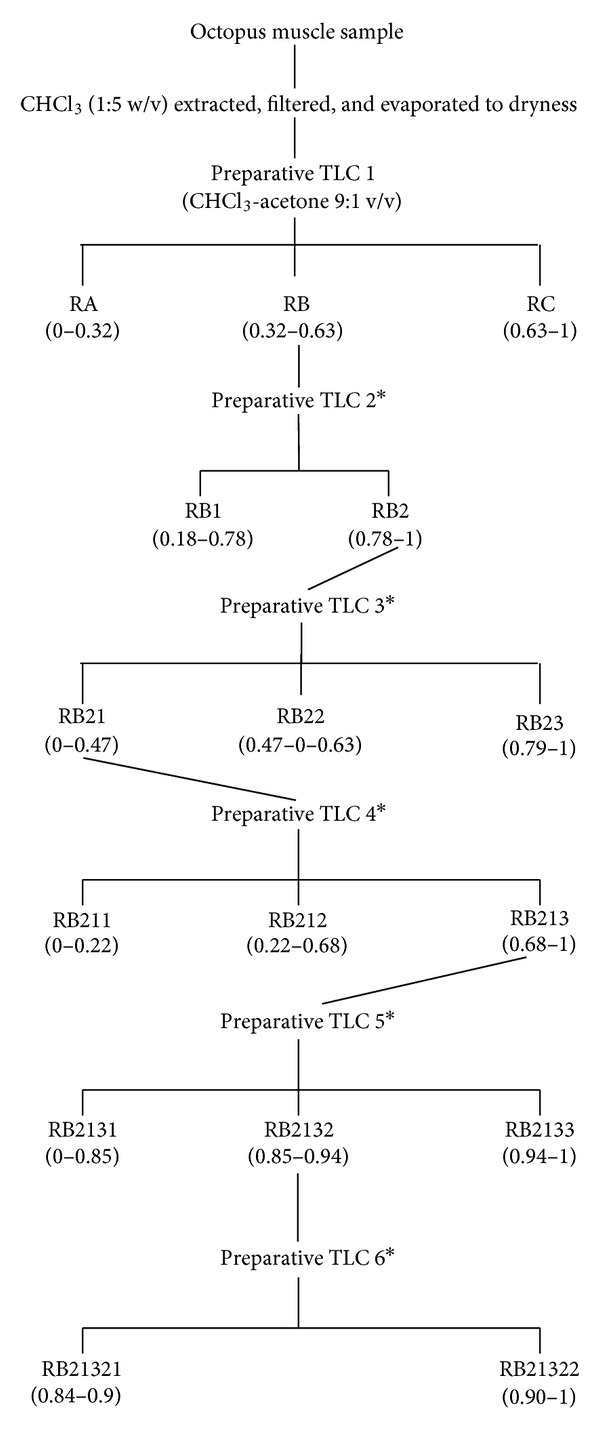
Schematic for separation and isolation of antimutagenic fractions from octopus. Numbers in parenthesis are *R*_*f*_ values. *TLC procedure conditions were identical to TLC1.

**Figure 2:**
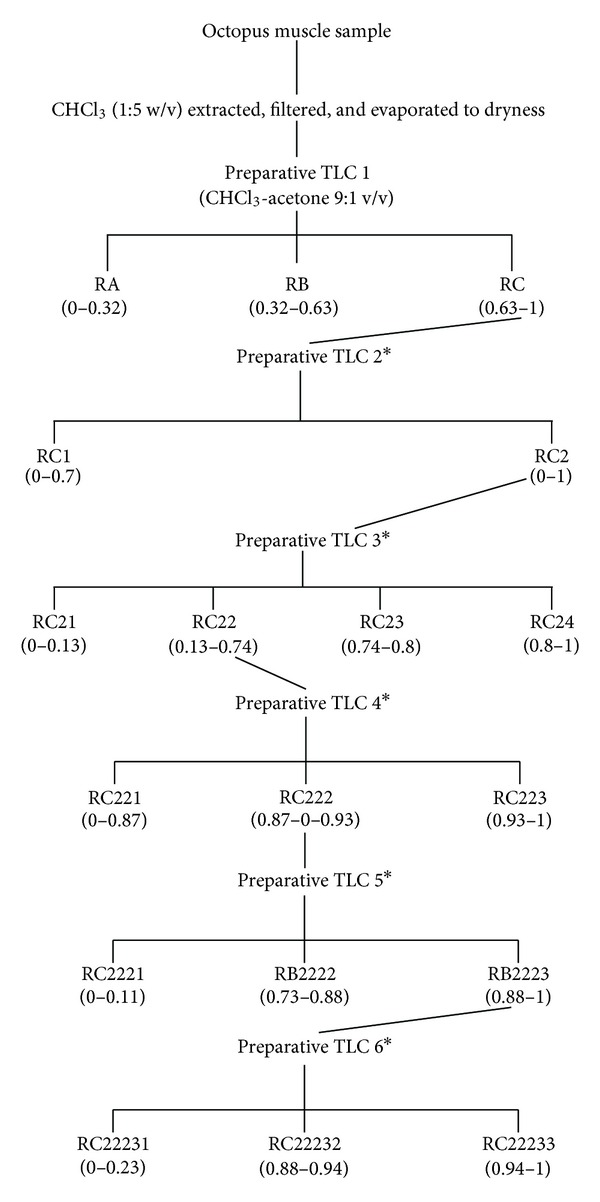
Schematic for separation and isolation of antiproliferative fractions from octopus. Numbers in parenthesis are *R*_*f*_ values. *TLC procedure conditions were identical to TLC1.

The fractionation of octopus extract was performed according to Burgos-Hernandez et al. [8]. A 2.0 mL aliquot of octopus muscle extract was applied on to a 1.0 mm thick silica gel-coated preparative TLC plate and developed with chloroform-acetone (9 : 1 v/v). Fluorescent bands, identified with their *R*_*f*_, were scrapped off the plate, and the contents of the silica were extracted with 2 × 25 mL chloroform-methanol-acetone (9 : 1 : 1 v/v/v). Extract were resuspended and serially diluted in DMSO and tested for antimutagenicity and antiproliferation. When either antimutagenic or antiproliferative (or both) bands were detected, their contents were obtained again from a fresh muscle sample following the same procedure and were subjected to further fractionation (Figures 1 and 2).

### 2.3. Bacterial Cultures

*Salmonella typhimurium* TA98 and TA100 were purchased from Molecular Toxicology Inc. Fresh overnight tester strain cultures, to which DMSO was added as cryoprotective agent, and were stored at −80°C. Tester strains were checked routinely to confirm genetic features using the procedure described by Maron and Ames [9]. 

*Metabolic Activation System*. S9 mix (Aroclor 1254-induced, Sprague-Dawley male rat liver in 0.154 M KCl solution) was purchased from Molecular Toxicology, Inc. (Annapolis, MD, USA) and stored at −80°C.

### 2.4. Antimutagenicity Test

Dry extracts obtained either directly from octopus or from preparative-TLC fractionation of extracts were reconstituted and serially diluted with DMSO and were spiked with pure AFB_1_ to a final concentration of 500 ng of AFB_1_/100 *μ*L.

Residual mutagenicity of AFB_1_ was assayed using the standard plate incorporation procedure described by Maron and Ames [9]. Different AFB_1_ concentrations were used as a control for both tester strains. All assays were performed in triplicate.

### 2.5. Cell Lines

Cell lines NCTC clone L929 (normal subcutaneous connective tissue) was purchased from the American Type Culture Collection (ATCC, Rockville, MD). The M12.C3.F6 (murine B-cell lymphoma) cell line was kindly provided by Dr. Emil R. Unanue (Department of Pathology and Immunology, Washington University in St. Louis, MO). All cell cultures were cultured in Dulbecco's modified Eagle's medium (DMEM) supplemented with 5% heat inactivated fetal calf serum and grown at 37°C in an atmosphere of 5% CO_2_.

### 2.6. Antiproliferation Assay

In order to evaluate the effect of octopus crude extracts and their fractions on the proliferation of different cancer cell lines, cell proliferation was determined using the standard MTT assay (3-(4,5-dimethylthiazol-2-yl)-2,5-diphenyltetrazolium bromide) [10]. Briefly, 10,000 cells (50 *μ*L) were plated in each well of a flat 96 well plate. After 12 h incubation at 37°C in an atmosphere of 5% CO_2_ to allow cell attachment, the cell cultures were incubated with 50 *μ*L of medium containing various concentrations of either crude extract or fraction, and the cell cultures were incubated for 48 h. The crude extract or fraction was first resuspended in DMSO and then diluted in DMEM media. Control cell cultures were incubated with DMSO (final concentrations of DMSO 0.06%–0.5%). Control cell cultures did not show any evidence of cell damage. In the last 4 h of the cell culture, 10 *μ*L of MTT stock solution (5 mg/mL) were added to each well [10]. Formazan crystals formed were dissolved with acidic isopropanol, and the plates were read in an ELISA plate reader (Benchmark Microplate Reader, Bio-Rad, Hercules, CA, USA), using a test wavelength of 570 nm and a reference wavelength of 630 nm. Plates were normally read within 15 min of adding isopropanol. Data were analyzed using analysis of variance (ANOVA) with Tukey-Kramer and Duncan's multiple comparison tests (Number Cruncher Statistical Software (NCSS 2000)).

### 2.7. Partial Chemical/Structural Studies

In order to know about aspects of the structural characteristics of the compounds present in the bioactive fractions from octopus, instrumental studies using Fourier Transformed Infrared (FT-IR) and nuclear magnetic resonance (^1^H-NMR) were carried out to attempt to contribute to a partial chemical structural characterization. To conduct FT-IR spectroscopy, thin films of the bioactive fractions samples were elaborated on ZnSe cells, and spectra were obtained using a GX Perkin Elmer equipment. For NMR spectra, bioactive fractions were dissolved in CDCl3 and analyzed in a Bruker ADVANCE 400 spectrometer using tetramethylsilane (TMS) as a reference. For mass spectrometry, contents of the antimutagenic fraction were analyzed using a Varian 431-GC gas chromatograph equipped with a VARIAN 210-MS ion trap mass detector. Chromatographic separation was achieved using a capillary column CP-SIL 43CB (25 m × 0.32 mm × 0.2 *μ*m) from VARIAN and an ultrahigh purity helium as carrier gas at a constant flow of 13 mL/min, in splitless injection mode. Three *μ*L of underivatized and derivatized with BF3/methanol sample were injected. An initial oven temperature of 120°C was held for 5 min, followed by a ramp of 10°C/min to 180°C, holding for 30 min, followed by a ramp of 10°C/min to 210°C, holding for 20 min. The injection port temperature was maintained at 250°C. Total chromatographic separation was achieved in 64 min. The ion trap detector was set as follows. The transfer line, manifold, and trap temperatures were 205, 80, and 150°C, respectively. All mass spectra were acquired in the electron impact mode. Ionization was maintained off during the first 2 min, to avoid solvent overloading. The mass range was 40–500 m/z, with a scan rate of 1 scan/s.

## 3. Results

### 3.1. Antimutagenicity

Octopus muscle was extracted and fractionated using TLC, and the fractions obtained (Figure 1) were tested for antimutagenicity. RA, RB, and RC fractions were obtained after the first TLC procedure (TLC 1). Antimutagenicity testing showed all extracts to have an inhibitory effect on the mutagenicity of 500 ng of AFB_1_ for both tester strains (Table 1) suggesting the presence of antimutagenic compounds in all of them; however, a well-defined dose-response type of relationship was observed for fraction RB, and it was selected for further fractionation since it was the fraction that achieved the greater inhibition (>76% for both tester strains) of the mutagenicity of induced by 500 ng AFB_1_. After second TLC isolation procedure, materials present in RB separated into 2 fractions (Figure 1). Fraction RB2 decreased the reversion rate achieved by 500 ng AFB_1_ (>75% for TA100 and >90% for TA98) suggesting that antimutagenic compounds were contained in this fraction (Table 2). Although other fractions also decreased reversion caused by AFB_1_, dose-response type of relationships was not as consistent in both tester strains as that observed for RB2. Therefore, RB2 was selected for further fractionation. After a third TLC isolation, procedure 3 fractions were obtained from RB2 (Figure 1). Antimutagenicity results showed that fraction RB21 differed from the rest since it effectively inhibited the mutagenicity of AFB_1_ induced in both tester strains (Table 3). RB21, which inhibited the mutagenic potential of AFB_1_ in more than 95% and 89% for TA98 and TA100 tester strains, respectively, was selected for further fractionation. Fractionation of RB21 (TLC 4) resulted into three regions, RB211, RB212, and RB213. Antimutagenesis testing revealed that fraction RB213 more (>66% of mutagenicity inhibition for both tester strains) efficiently decreased tester strains reversion rate than RB211 and RB212 (both <40% for both TA98 and TA100), showing a dose-response relationship with a lower slope (Table 4).

Therefore, RB213 was selected and fractionated by means of a TLC 5 procedure. Development of TLC 5 showed that most of the materials migrated to the top half portion of the plate. This allowed the determination of 3 fractions that were named RB2131 (no materials observed), RB2132, and RB2133; therefore, only de 2 top fractions were assayed.

The antimutagenicity assay performed showed RB2132 as the fraction that inhibited the mutagenicity of AFB_1_ in more than 84% for both tester strains (Table 5), whereas RB2133 inhibitory activity was no higher than 40%; therefore, RB2132 contents were fractionated using a sixth TLC procedure. TLC 6 applied to RB2132 resulted in only 2 bands, RB21321 and RB21322. The contents form these bands were extracted and tested for antimutagenicity. Results from this assay showed that both bands contained compounds that inhibited the mutagenicity of AFB_1_ in a dose-response type of relationship (Table 6). RB21321 inhibited more than 70% the mutagenicity of AFB_1_ in both tester strains, whereas RB21322 exhibited an inhibition activity higher than 55% for both TA98 and TA100 tester strains. The contents from both bands were subjected for further TLC procedures using different solvent system without successful fractionation.

### 3.2. Antiproliferation Activity

**Figure 3:**
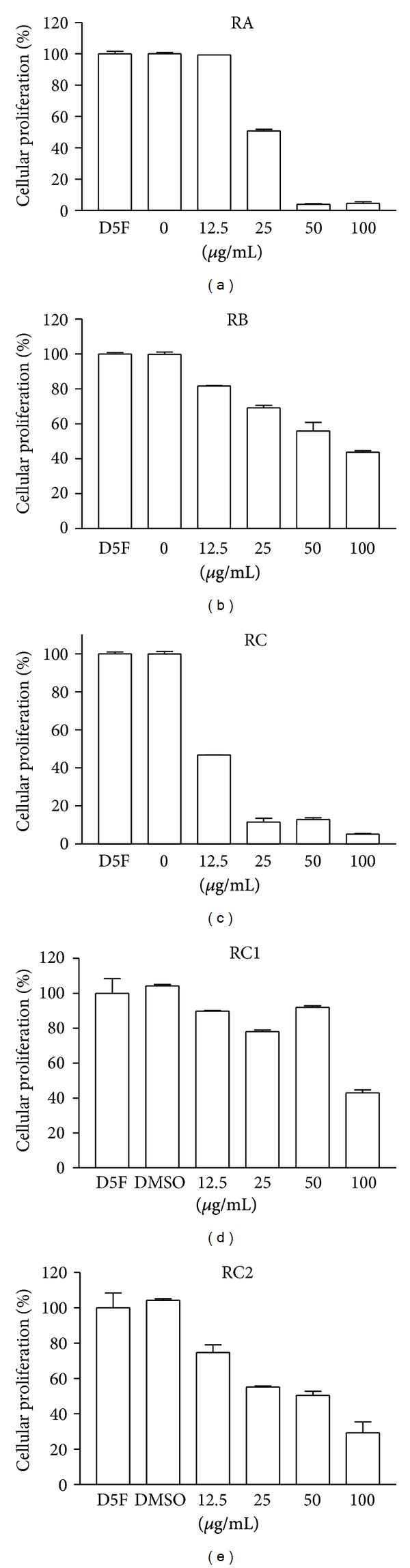
Antiproliferative effect of lipid extracts from octopus on murine cancerous cell lines. Murine cancer M12.C3.F6 cell lines were treated with different dose lipidic extracts during 48 h. Cellular proliferation was determined by standard MTT assay (3-(4,5-dimethylthiazol-2-yl)-2,5-diphenytetrazolium bromide). The results shown are representative from at least three independent experiments. All values represent mean of triplicate determination ± standard deviation. Significant differences (*P* ≤ 0.05) from control cell cultures are marked with an asterisk. Control cell cultures were incubated with DMSO (0.5%).

In order to investigate the presence of antiproliferative agents in octopus lipidic extract, a fractionation procedure parallel to that carried out for antimutagens was performed (Figure 2). The fractions RA, RB, and RC obtained from the first TLC fractionation were tested on the antiproliferative assays. All octopus fractions showed antiproliferative activity on the murine cancer cell line M12.C3.F6 (B-cell lymphoma) in a concentration-dependent manner (Figure 3). However, only fractions RA and RC were able to inhibit the cellular proliferation beyond 50%, at the lowest doses tested (12.5 and 25 *μ*g/mL). The highest level of cellular proliferation inhibition was observed for fraction RC (about 90% for the second lowest dose tested (25 *μ*g/mL); therefore, RC was selected for further fractionation.

From the second TLC fractionation step, fractions RC1 and RC2 were obtained (Figure 2) and tested for antiproliferation activity (Figure 3). Fractions RC1 and RC2 were able to inhibit cellular proliferation in more than 50% at the highest dose tested (100 *μ*g/mL). However, more than 60% cellular proliferation inhibition was observed in cancer cell cultures exposed to fraction RC2; therefore, this fraction was selected to continue with the fractionation process.

**Figure 4:**
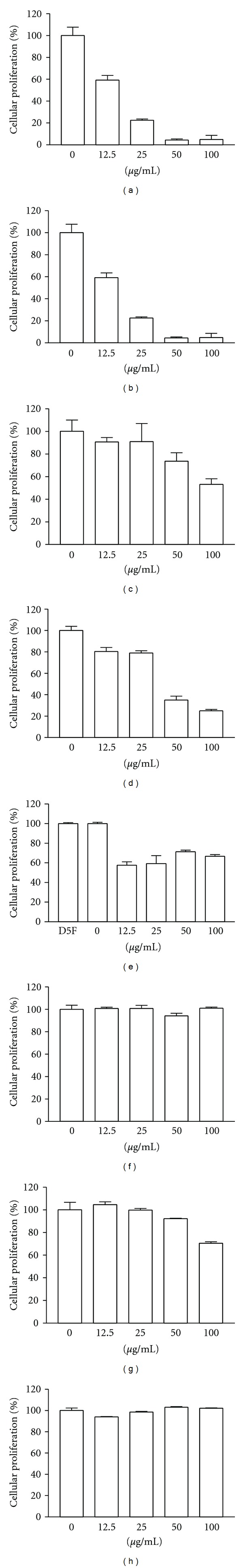
Antiproliferative effect of lipid extracts from octopus on murine cancerous and noncancerous cell lines. Murine cancer M12.C3.F6 (a)–(d) and noncancerous (e)–(h) cell lines were treated with different dose lipidic extracts during 48 h. The results shown are representative from at least three independent experiments. All values represent mean of triplicate determination ± standard deviation. Significant differences (*P* ≤ 0.05) from control cell cultures are marked with an asterisk. Control cell cultures were incubated with DMSO (0.5%).

Figures 4(a)–4(d) show the antiproliferative activities of octopus fractions obtained from subsequent TLC fractionation (TLC 3, TLC 4, TLC 5, and TLC 6) of fraction RC2. All fractions derived from RC2 had significant inhibitory effect on the growth of the cancer cell line tested. In contrast, RC2-derived fractions showed a much lower antiproliferative effect on the murine noncancerous cell line L-929 than on the cancer cell line M12.C3.F6 (Figures 4(e)–4(h)).

### 3.3. Partial Chemical/Structural Studies

**Figure 5:**
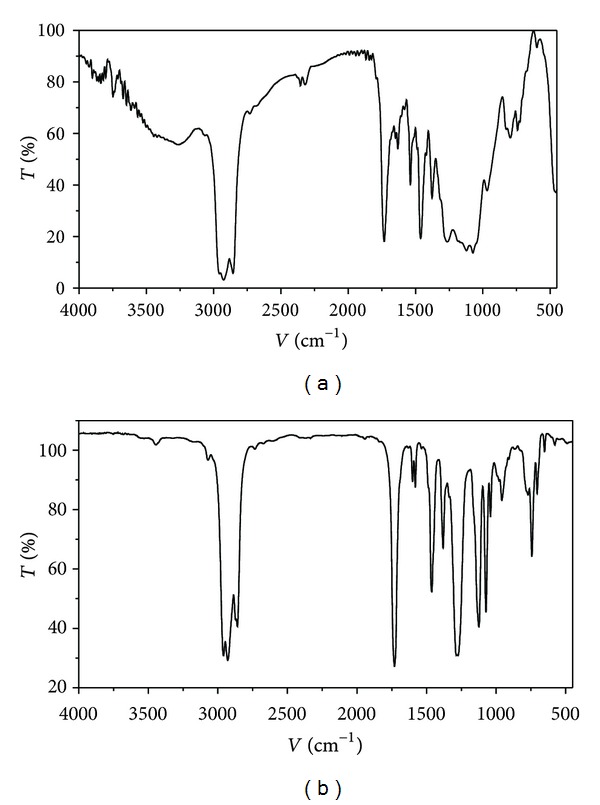
FT-IR spectra of antimutagenic fraction RB21321 (a) and antiproliferative fraction RC22233 (b), both obtained from a lipidic extract from octopus.

**Figure 6:**
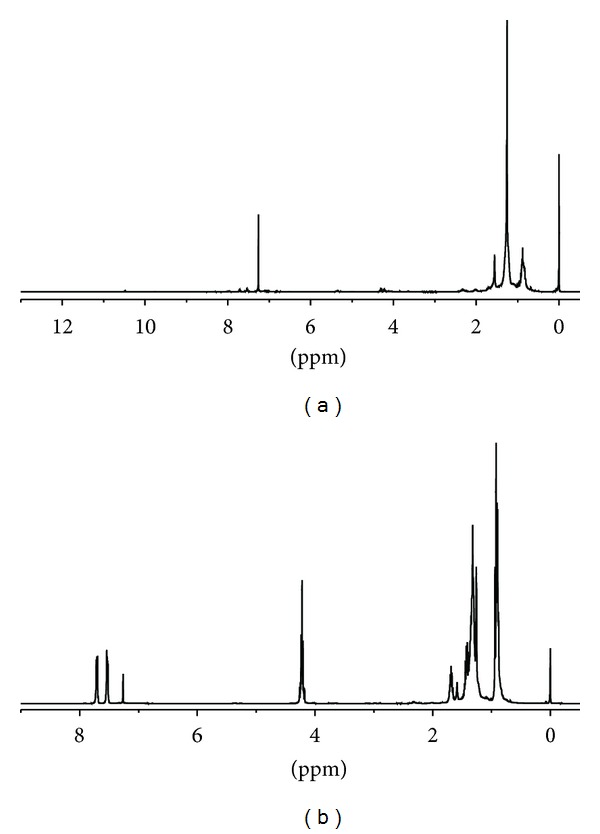
^1^H-RMN spectra (CDCl3/TMS) of antiproliferative fraction RC22233 (a) and antimutagenic fraction RB21321 (b), both obtained from a lipidic extract from octopus.

In order to obtain information about the presence of functional groups and structural aspects to the materials detected in the bioactive (both, antimutagenic fraction RB21321 and antiproliferative fraction RC22233) isolated fractions from octopus, FT-IR and ^1^H-NMR analyses were performed. NMR (Figure 5) studies showed broad peaks visualized in the 1.3 ppm region; these signals were attributed to methylene protons, probably from long chain fatty acids, such as those present in *ω*-3 fatty acids. Additional contributions obtained from lipid resonances (0.9–1.7 ppm region) are also present. In this, the wide peak at 0.9 ppm region was associated to methyl groups [11]. Results from FT-IR (Figure 6) carried out on bioactive fractions from octopus provided of a spectra that coincided in a typical signal for carbonyl function group; the peaks visualized in the 1600–1680 range could be related to the unsaturated hydrocarbons featuring C=C, with attached hydrogens. The C–H stretch vibrations at 2900 cm^−1^ may be attributed to methylene groups; this agrees with results from NMR which suggest the presence of fatty acids. However, the signals observed in the aromatic region (7.5–7.8 ppm) of the ^1^H-NMR spectra suggest the possibility of the presence of an aromatic type of compound or a compound with a cyclic portion in its structure.

**Figure 7:**
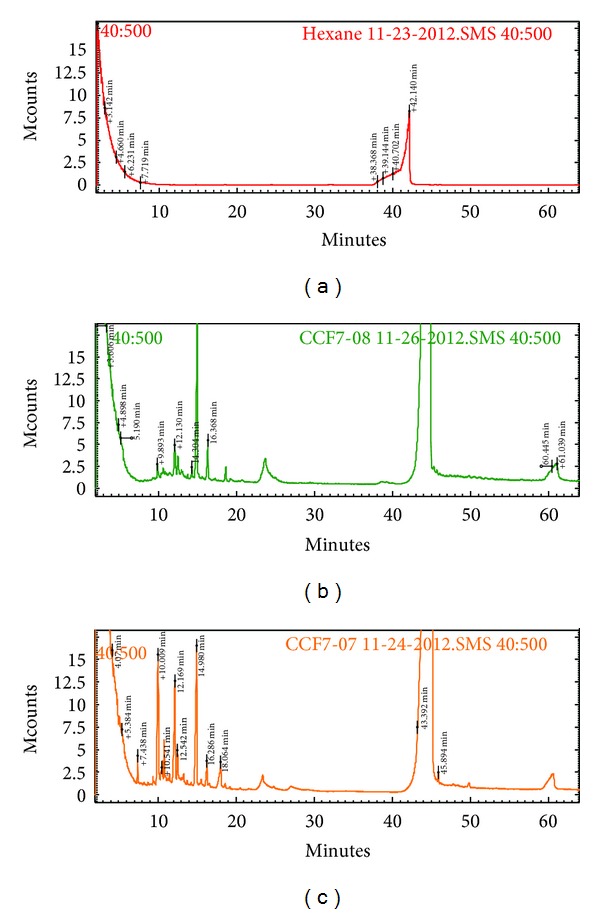
GC-MS spectra of underivatized (b) and derivatized antimutagenic fraction RB21321 (c) obtained from the lipidic fraction of octopus. Samples were solubilized in hexane (a).

Antimutagenic fraction RB21321 was analyzed by mass spectrometry. Mass spectrum (Figure 7) shows 29 peaks with retention times ranging from 7 to 61 min. Most of these peaks showed retention times shorter than 20 min. When the contents of the antimutagenic fraction were derivatized with BF3/methanol, 5 additional peaks were observed within this interval, which correspond to methylated long-chain fatty acids. This confirms the results obtained from the RMN and FT-IR analyses. Even though the antimutagenic fraction analyzed is still a mixture of several compounds (possibly saturated and unsaturated fatty acids), more than 70% of the fraction consists of only 5 different compounds. This is in accordance with the signs obtained by NMR results. However, further investigation is necessary to conduct for a full structural characterization.

## 4. Discussion

Previous research performed in our laboratory allowed us to detect the existence of compounds in yellowtail fish (*Seriola lalandi*), lisa fish (*Mugil cephalus*), and cazon fish (*Mustelus lunulatus*) [8], as well as in shrimps [12] that have the capability to inhibit the mutagenicity of aflatoxin B_1_ in the Ames test. Many substances found in the marine environment have been associated to a number of biological properties. Omega-3 polyunsaturated fatty acids (*ω*-3PUFAs) have been implicated in chemoprevention of cancer [5, 13, 14]. Omega-3 PUFAs also have been related to the suppression of cancerous tumors [15, 16] and to reduced risk for prostate cancer [17, 18]. Therefore, the present research work intended to isolate compound(s) from the lipidic fraction of octopus that would have antimutagenic and/or antiproliferative properties. All of the fractions obtained right after the first fractionation procedure were capable to inhibit the induction of bacterial reversion, showing a dose-response type of relationship in both tester strains, suggesting the presence of antimutagens in every fraction. All of the fractions were able to cause an inhibition of AFB_1_ mutagenicity close to 50%, even at the highest dilution tested. At this point, we could have selected any of the fractions to continue with the isolation process. However, for both strains, the lowest reversion rate was observed for fraction RB, suggesting that contents from this fraction might have a higher potency in inhibiting the mutagenicity of AFB_1_; therefore, RB was selected for further fractionation.

Additional studies on the antimutagenic properties of fractions RA and RC are undergoing.

Fraction RB was fractionated into 2 groups of bands coded as RB1 and RB2 (Figure 1). Antimutagenesis testing performed to the extracts from these fractions (Table 2) showed that fraction RB2 had the most consistent dose-response type of relationship in both tester strains. RB1 partially inhibited the mutagenicity of AFB_1_ towards both tester strains; however, it failed to show a dose-response type of relationship. However, these results may suggest the presence of antimutagenic compounds which are minor components of fraction RB or have less antimutagenic potency than those contained in fractions RB2. Based on the dose-response relationship and antimutagenic potency, fraction RB2 was selected for further fractionation.

Fraction RB2 was separated into 3 bands which were also tested for antimutagenicity (Table 3). From the three extracts obtained and tested, fraction RB21 caused the highest inhibition of AFB_1_ mutagenicity showing a dose-response type of relationship and lowering the reversion rate at the spontaneous reversion when was undiluted. Fractions RB22 and RB23 showed antimutagenicity when undiluted; however, this activity was lost more rapidly upon first dilution. A possible reason for this apparent loss of antimutagenicity after the third step in the fractionation process is the possible existence of synergistic antimutagenic compounds that may lose their antimutagenic properties when are separate. However, the possible loss of material throughout the fractionation process is not discounted. Based on the aforementioned, the fractionation of RB21 proceeded. The contents of RB21 fractionated into three regions on the TLC plate: RB211, RB212, and RB213. Results from the antimutagenicity assay showed that RB212 inhibited AFB_1_ in a greater extent than the other two fractions. These results suggested that antimutagenic compounds were concentrated in this fraction located in the middle portion of the TLC plate. The contents of this fraction were extracted and subjected to a fifth TLC procedure in order to continue with the isolation of antimutagenic compounds from octopus. After TLC 5, materials contained in fraction RB213 migrated to the top portion of the plate. In order to facilitate the extraction of the materials, the plate was divided into 3 fractions, RB2131 (no materials observed), RB2132, and RB213. Although both fractions RB2132 and RB213 showed antimutagenic potential, contents from RB2132 inhibited AFB_1_ mutagenicity in both tester strains showing a consistent dose-response type of relationship. The sixth TLC procedure applied to RB2132 resulted in two other fractions, RB21321 and RB21322. Contents from both fractions were localized in the upper portion of the plate, showing their affinity for the solvent used (CHCl_3_-acetone 9 : 1). Both were antimutagenic showing similar dose-response relationships, which suggest the presence of at least 2 compounds with similar antimutagenic characteristics. Attempts to fraction both RB21321 and RB21322 using different solvents system were unsuccessful, suggesting that the chemical nature of the compounds contained in both fractions is similar. Further studies on the chemical/structural characterization of RB21321 and RB21322 are under way.

As mentioned before, three regions (RA, RB, and RC) were obtained from the fractionation of the lipidic extract from octopus. Fraction RB was the most antimutagenic fraction; however, all fractions showed antiproliferative activity, being RA and RC the fractions that had the highest inhibition of cancer cell proliferation (Figure 3). These results suggest that all three fractions contained both antimutagens and antiproliferative compounds; however, this investigation focused on the search for the compounds with the most potent activities. Fractions RA and RC inhibited murine cancer cell proliferation in a dose-dependent manner; however, at the lowest dose tested (12.5 *μ*g/mL), fraction RC inhibited more than 50% of cell proliferation compared to the control. Therefore, RC was considered the most potent antiproliferative fraction, and it was selected for further fractionation.

Two fractions are derived from fraction RC (RC1 and RC2). RC2, at a dose of 100 *μ*g/mL, inhibited more than 60% of cell proliferation compared to the control (Figure 3). These results suggest that antiproliferative compounds of fraction RC were partitioned to fraction RC2; therefore, this fraction was subjected to additional TLC procedures.

From the third TLC fractionation step, three fractions (RC21, RC22, and RC23) were obtained from fraction RC2; fraction RC22 showed the highest inhibition of cancer cell proliferation (close to 90%). This might suggest that components with this type of biological activity were isolated into this fraction and possibly separated from other interfering compounds. Along with the fractionation procedure (similar to that followed to isolate antimutagenic compounds), several fractions were obtained from which the presence of antiproliferative compounds was evident (Figure 4). These lipidic compounds present in those fractions from octopus substantially delayed the growth of the murine cancer cell M12.C3.F6, but the growth of normal murine cell line L-929 was considerably less affected. These observations suggest that active constituents of octopus have preferential antiproliferative effects on cancer cell lines.

## 5. Conclusion

Further studies need to be performed for a full chemical and biological characterization of the preferential effect of octopus fractions on murine cancer cell lines. Results from this study suggest that within various compounds in the lipid fraction of octopus, a group of saturated and unsaturated fatty acids are responsible for the bioactivity against either the mutagenicity of AFB_1_ or murine cancerous cell proliferation, or against both. Although n-3PUFAs may be considered as factors partially responsible for the bioactivities observed in the first fractions obtained in this study, the isolation and identification of the actual antimutagenic and antiproliferative compounds in octopus are the focus of our ongoing research.

## Figures and Tables

**Table 1:** Antimutagenic potential of extracts from fractions RA, RB, and RC obtained from lipidic extract of octopus (average^a^ TA100 and TA98 revertants/plate, with S9).

Extract fraction^c^	Fraction dilution^c^			
	1 × 10^0^	1 × 10^−1^	1 × 10^−2^	1 × 10^−3^
TA98				
RA (*R*_*f*_ = 0.0–0.29)	170 ± 13	296 ± 24	267 ± 13	333 ± 18
RB (*R*_*f*_ = 0.29–0.69)	117 ± 10	271 ± 18	338 ± 27	350 ± 31
RC (*R*_*f*_ = 0.69–1.0)	215 ± 7	387 ± 23	299 ± 21	453 ± 33

TA100				
RA (*R*_*f*_ = 0.0–0.29)	71 ± 16	203 ± 12	567 ± 35	621 ± 53
RB (*R*_*f*_ = 0.29–0.69)	127 ± 3	361 ± 39	534 ± 37	567 ± 24
RC (*R*_*f*_ = 0.69–1.0)	185 ± 15	565 ± 43	523 ± 48	536 ± 30

^a^AFB_1_ (used as positive control) tested at 100, 500, and 1000 ng/plate induced 195 ± 23, 495 ± 29, and 676 ± 55 and 200 ± 16, 595 ± 43, and 745 ± 24 revertants/plate for TA98 and TA100, respectively. Spontaneous revertants were 55 ± 7 and 113 ± 19 for TA98 and TA100, respectively.

^b^Values are means of three replicates ± SEM.

^c^Extracts were diluted and spiked with enough toxin to yield 500 ng of pure AFB_1_/plate.

**Table 2:** Anti-mutagenic potential of AFB_1_^a^-spiked fractions obtained from thin layer chromatography fractionation of fraction RB (*R*_*f*_ = 0.18–0.78) from TLC 1 (average^b^ TA100 and TA98 revertants/plate, with S9).

Extract fraction^c^	Fraction dilution^c^			
	1 × 10^0^	1 × 10^−1^	1 × 10^−2^	1 × 10^−3^
TA98				
RB1 (*R*_*f*_ = 0.18–0.78)	464 ± 98	1230 ± 64	933 ± 59	1023 ± 53
RB2 (*R*_*f*_ = 0.78–1.00)	82 ± 13	548 ± 48	632 ± 56	955 ± 65

TA100				
RB1 (*R*_*f*_ = 0.18–0.78)	564 ± 37	1355 ± 94	1299 ± 105	1283 ± 88
RB2 (*R*_*f*_ = 0.78–1.00)	315 ± 20	348 ± 25	668 ± 32	740 ± 13

^a^AFB_1_ (used as positive control) tested at 500 and 1000 ng/plate induced 1073 ± 29 and 1841 ± 102 and 1229 ± 37 and 2286 ± 103 revertants/plate for TA98 and TA100, respectively. Spontaneous revertants were 52 ± 4 and 160 ± 4 for TA98 and TA100, respectively.

^b^Values are means of three replicates ± SEM.

^c^Extracts were diluted and spiked with enough toxin to yield 500 ng of pure AFB_1_/plate.

**Table 3:** Anti-mutagenic potential of AFB_1_^a^ -spiked fractions obtained from thin layer chromatography fractionation of fraction RB2 (*R*_*f*_ = 0.78–1.00) from TLC 2 (average^b^ TA100 and TA98 revertants/plate, with S9).

Extract fraction^c^	Fraction dilution^c^			
	1 × 10^0^	1 × 10^−1^	1 × 10^−2^	1 × 10^−3^
TA98				
RB21 (*R*_*f*_ = 0.00–0.47)	49 ± 9	73 ± 4	257 ± 20	325 ± 24
RB22 (*R*_*f*_ = 0.47–0.79)	71 ± 3	141 ± 20	219 ± 19	218 ± 28
RB23 (*R*_*f*_ = 0.79–1.00)	18 ± 2	175 ± 13	236 ± 18	265 ± 21

TA100				
RB21 (*R*_*f*_ = 0.00–0.47)	175 ± 30	198 ± 18	220 ± 15	275 ± 71
RB22 (*R*_*f*_ = 0.47–0.79)	292 ± 12	455 ± 31	843 ± 27	883 ± 12
RB23 (*R*_*f*_ = 0.79–1.00)	320 ± 25	695 ± 40	567 ± 44	1687 ± 70

^a^AFB_1_ (used as positive control) tested at 500 ng/plate induced 1006 ± 83 and 1689 ± 78 revertants/plate for TA98 and TA100, respectively. Spontaneous revertants were 55 ± 3 and 188 ± 14 for TA98 and TA100, respectively.

^b^Values are means of three replicates ± SEM.

^c^Extracts were diluted and spiked with enough toxin to yield 500 ng of pure AFB_1_ plate.

**Table 4:** Anti-mutagenic potential of AFB_1_^a^-spiked fractions obtained from thin layer chromatography fractionation of fraction RB21 (*R*_*f*_ = 0.78–1.00) from TLC 2 (average^b^ TA100 and TA98 revertants/plate, with S9).

Extract fraction^c^	Fraction dilution^c^			
	1 × 10^0^	1 × 10^−1^	1 × 10^−2^	1 × 10^−3^
TA98				
RB211 (*R*_*f*_ = 0.00–0.22)	628 ± 49	576 ± 43	499 ± 55	549 ± 34
RB212 (*R*_*f*_ = 0.22–0.68)	371 ± 13	345 ± 25	489 ± 29	650 ± 68
RB213 (*R*_*f*_= 0.68–1.00)	199 ± 15	335 ± 23	401 ± 28	576 ± 21

TA100				
RB211 (*R*_*f*_ = 0.00–0.22)	1549 ± 89	1466 ± 123	1657 ± 220	1425 ± 124
RB212 (*R*_*f*_ = 0.22–0.68)	1006 ± 93	941 ± 45	1178 ± 129	918 ± 28
RB213 (*R*_*f*_ = 0.68–1.00)	499 ± 35	829 ± 63	1237 ± 128	1465 ± 121

^a^AFB_1_ (used as positive control) tested at 500 ng/plate induced 605 ± 33 and 1689 ± 78 revertants/plate for TA98 and TA100, respectively. Spontaneous revertants were 55 ± 3 and 188 ± 14 for TA98 and TA100, respectively.

^b^Values are means of three replicates ± SEM.

^c^Extracts were diluted and spiked with enough toxin to yield 500 ng of pure AFB_1_/plate.

**Table 5:** Anti-mutagenic potential of AFB_1_^a^-spiked fractions obtained from thin layer chromatography fractionation of fraction RB213 (*R*_*f*_ = 0.68–1.00) from TLC 2 (average^b^ TA100 and TA98 revertants/plate, with S9).

Extract fraction^c^	Fraction dilution^c^			
	1 × 10^0^	1 × 10^−1^	1 × 10^−2^	1 × 10^−3^
TA98				
RB2132 (*R*_*f*_ = 0.85–0.94)	79 ± 11	245 ± 25	409 ± 29	502 ± 28
RB2133 (*R*_*f*_ = 0.94–1.00)	399 ± 35	435 ± 49	489 ± 41	576 ± 21

TA100				
RB2132 (*R*_*f*_ = 0.85–0.94)	178 ± 13	389 ± 41	603 ± 59	918 ± 28
RB2133 (*R*_*f*_ = 0.94–1.00)	499 ± 35	829 ± 63	1237 ± 128	1465 ± 121

^a^AFB_1_ (used as positive control) tested at 500 ng/plate induced 491 ± 33 and 1283 ± 78 revertants/plate for TA98 and TA100, respectively. Spontaneous revertants were 54 ± 3 and 128 ± 11 for TA98 and TA100, respectively.

^b^Values are means of three replicates ± SEM.

^c^Extracts were diluted and spiked with enough toxin to yield 500 ng of pure AFB_1_/plate.

**Table 6:** Anti-mutagenic potential of AFB_1_^a^-spiked fractions obtained from thin layer chromatography fractionation of fraction RB2132 (*R*_*f*_ = 0.68–1.00) from TLC 2 (average^b^ TA100 and TA98 revertants/plate, with S9).

Extract fraction^c^	Fraction dilution^c^			
	1 × 10^0^	1 × 10^−1^	1 × 10^−2^	1 × 10^−3^
TA98				
RB21321 (*R*_*f*_ = 0.84–0.90)	169 ± 11	263 ± 17	461 ± 29	534 ± 48
RB21322 (*R*_*f*_ = 0.90–1.00)	150 ± 14	301 ± 29	423 ± 38	401 ± 45

TA100				
RB21321 (*R*_*f*_ = 0.84–0.90)	278 ± 23	492 ± 41	603 ± 59	918 ± 28
RB21322 (*R*_*f*_ = 0.90–1.00)	423 ± 39	734 ± 52	847 ± 78	907 ± 37

^a^AFB_1_ (used as positive control) tested at 500 ng/plate induced 536 ± 39 and 958 ± 88 revertants/plate for TA98 and TA100, respectively. Spontaneous revertants were 20 ± 4 and 120 ± 9 for TA98 and TA100, respectively.

^b^Values are means of three replicates ± SEM.

^c^Extracts were diluted and spiked with enough toxin to yield 500 ng of pure AFB_1_/plate.
